# Supplementary material for: Cadmium and volumetric mammographic density: A cross-sectional study in Polish women
Source: PLoS One. 2020 May 20;15(5):e0233369. doi: 10.1371/journal.pone.0233369 (PMC7239444; doi:10.1371/journal.pone.0233369)
Supplement: S1 Table — (DOCX) [file pone.0233369.s001.docx]

S1_Table. Association between urinary cadmium concentration and percent volumetric mammographic density and fibroglandular tissue volume

|  |  | Estimate (95% Confidence interval), regression of log outcome on log cadmium concentration | | | |
| --- | --- | --- | --- | --- | --- |
| Outcome |  | unadjusted | Adjusted^1^ | Adjusted^2^ | |
| Percent volumetric breast density | Coef. β^3^  exp(β)^4^  Ratio of VBD per doubling of Cr-adj. Cd^5^ | -0.038 (-0.114,0.038)  0.963 (0.893,1.038)  0.974 (0.924,1.026) | **-0.075 (-0.140,-0.010)**  **0.928 (0.869,0.990)**  **0.949 (0.908,0.993)** | -0.055 (-0.123,0.012)  0.946 (0.884,1.012)  0.962 (0.918,1.008) | |
| Fibroglandular tissue volume | Coef. β^3^  exp(β)^4^  ratio of FG per doubling of Cr-adj. Cd^5^ | -0.039 (-0.105,0.028)  0.962 (0.900,1.028)  0.973 (0.930,1.019) | -0.016 (-0.081,0.048)  0.984 (0.923,1.049)  0.989 (0.946,1.034) | -0.003 (-0.070,0.064)  0.997 (0.933,1.067)  0.998 (0.953,1.046) | |
|  |  | Mean (95%Confidence interval)percent volumetric mammographic density | | | |
| Cadmium quartile  Q1:[0.004,0.266]  Q2:(0.266,0.448]  Q3:(0.448,0.691]  Q4:(0.691,3.16] |  | 7.1 (6.5,7.9)  6.6 (6.1,7.3)  7.2 (6.6,7.9)  6.1 (5.5,6.7) | 7.4 (6.7,8.2)  6.7 (6.1,7.4)  7.0 (6.4,7.8)  6.2 (5.6,6.9) | | 7.2 (6.5,8.0)  6.6 (6.0,7.3)  7.0 (6.3,7.7)  6.3 (5.7,7.0) |
|  |  | Mean (95%Confidence interval) fibroglandular tissue volume (cm^3^) | | | |
| Cadmium quartile  Q1:[0.004,0.266]  Q2:(0.266,0.448]  Q3:(0.448,0.691]  Q3:(0.448,0.691] |  | 58.2 (53.4,63.5)  50.4 (46.7,54.5)  55.5 (51.3,60.0)  53.6 (49.2,58.5) | 57.8 (52.3,63.9)  49.6 (45.2,54.4)  56.1 (51.1,61.6)  53.5 (48.5,59.0) | | 56.9 (51.3,63.0)  49.0 (44.6,53.8)  55.9 (50.8,61.4)  53.9 (48.8,59.5) |

^1^ Adjusted for age at mammography; BMI; family breast cancer; mammographic device; season of the year of mammography; and age at menarche, creatinine

^2^ Adjusted for age at mammography; BMI; family breast cancer; mammographic device; season of the year of mammography; age at menarche, creatinine and smoking

^3^ Beta (β) coefficient for regression of log outcome on log Cd

^4^ exp(β) for regression of outcome on Cd, which corresponds to the ratio of geometric mean outcome associated with a unit increased in log Cd

^5^ exp(log(2)*β)
