# Supplementary material for: Cadmium and volumetric mammographic density: A cross-sectional study in Polish women
Source: PLoS One. 2020 May 20;15(5):e0233369. doi: 10.1371/journal.pone.0233369 (PMC7239444; doi:10.1371/journal.pone.0233369)
Supplement: S2 Table — (DOCX) [file pone.0233369.s002.docx]

S2­_Table. Association between cadmium concentration creatinine adjusted in urine and percent volumetric mammographic density and fibroglandular tissue volume by pregnancy status

|  | Parous women  n=432 | | | Nulliparous women  n=32 | | | p-heterogeneity^3^ |
| --- | --- | --- | --- | --- | --- | --- | --- |
|  | β (95%Confidence interval) | | | β (95%Confidence interval) | | |  |
|  | unadjusted | Adjusted^1^ | Adjusted^2^ | unadjusted | Adjusted^1^ | Adjusted^2^ |  |
| Percent volumetric mammographic density | -0.061 (-0.142, 0.021) | **-0.099 (-0.171, -0.028)** | **-0.087 (-0.160, -0. 013)** | 0.220 (-0.136, 0.575) | 0.038 (-0.355, 0.432) | 0.156 (-0.280, 0.591) | 0.068^1^  0.048^2^ |
| Fibroglandular tissue volume | **-0.081 (-0.155, -0.008)** | -0.042 (-0.114, 0.032) | -0.029 (-0.104, 0.046) | **0.277 (0.080, 0.473)** | 0.320 (0.044, 0.595) | **0.419 (0.122, 0.716)** | 0.022^1^  0.015^2^ |
|  | Mean (95%Confidence interval) percent volumetric mammographic density | | | | | |  |
| Cadmium quartile  Q1:[0.008,0.38]  Q2:(0.38,0.57]  Q3:(0.57,0.79]  Q4:(0.79,3.4] | 7.2 (6.5, 7.9)  7.4 (6.7, 8.2)  6.0 (5.5, 6.6)  6.9 (6.3, 7.6) | 7.9 (7.0, 9.0)  8.2 (7.2, 9.4)  6.8 (5.9, 7.7)  7.1 (6.2, 8.0) | 7.9 (6.2, 10.2)  8.3 (6.4, 10.6)  6.9 (5.3, 8. 8)  7.2 (5.6, 9.1) | 6.4 (4.0, 10.2)  5.5 (3.6, 8.3)  7.7 (4.6, 12.8)  7.7 (5.2, 11.4) | 6.0 (3.5, 10.3)  7.0 (4.3, 11.2)  9.0 (4.9, 16.6)  6.6 (4.6, 9.6) | 6.1 (3.4, 10.8)  7.6 (4.5, 12.8)  9.2 (4.8, 17.7)  7.6 (4.6, 12.6) |  |
|  | Mean (95%Confidence interval) fibroglandular tissue volume (cm^3^) | | | | | |  |
| Cadmium quartile  Q1:[0.008,0.38]  Q2:(0.38,0.57]  Q3:(0.57,0.79]  Q4:(0.79,3.4] | 56.4 (51.8, 61.4)  62.0 (56.9, 67.6)  52.0 (47.8, 56.6)  51.2 (46.9, 55.9) | 57.4 (50.5, 65.3)  62.3 (54.5, 71.1)  54.7 (47.8, 62.5)  55.5 (48.7, 63.4) | 61.4 (47.8, 78.8)  66.6 (51.7, 85.9)  58.9 (45.7, 75.9)  60.2 (47.1, 76.9) | 42.9 (33.13, 55.6)  53.9 (42.93, 67.8)  67.8 (51.3, 89.6)  65.4 (52.7, 81.2) | 40.1 (25.3, 63.6)  60.6 (40.7, 90.3)  74.6 (44.7, 124.4)  62.9 (46.0, 86.2) | 40.9 (25.6, 65.3)  66.4 (43.3, 101.8)  76.0 (44.5, 129.8)  72.6 (47.9, 109.9) |  |

^1^ Adjusted for age at mammography, BMI, family breast cancer, mammographic device, season of the year of mammography, and age at menarche

^2^ Adjusted for age at mammography, BMI, family breast cancer, mammographic device, season of the year of mammography, age at menarche and smoking

^3^  likelihood ratio test
