# Supplementary material for: Cadmium and volumetric mammographic density: A cross-sectional study in Polish women
Source: PLoS One. 2020 May 20;15(5):e0233369. doi: 10.1371/journal.pone.0233369 (PMC7239444; doi:10.1371/journal.pone.0233369)
Supplement: S3 Table — (DOCX) [file pone.0233369.s003.docx]

S3_Table. Association between cadmium concentration creatinine adjusted in urine and percent volumetric mammographic density and fibroglandular tissue volume by smoking

|  | Never smoking  N=226 | | Ex-smoker  N=137 | | Current smoker  N=110 | | p-heterogeneity^2^ |
| --- | --- | --- | --- | --- | --- | --- | --- |
|  | β (95%Confidence interval) | | β (95%Confidence interval) | | β (95%Confidence interval) | |  |
|  | unadjusted | Adjusted^1^ | unadjusted | Adjusted^1^ | unadjusted | Adjusted^1^ |  |
| Percent volumetric mammographic density | 0.005 (-0.114, 0.123) | -0.065 (-0.168, 0.037) | -0.124 (-0.260, 0.012) | -0.084 (-0.220, 0.052) | 0.010 (-0.168, 0.189) | -0.117 (-0.271, 0.036) | 0.791^1^ |
| Fibroglandular tissue volume | -0.039 (-0.141,0.064) | -0.005 (-0.107,0.096) | -0.038 (-0.161,0.085) | 0.059 (-0.074,0.192) | -0.111 (-0.273,0.051) | -0.080 (-0.246,0.085) | 0.651^1^ |

^1^ Adjusted for age at mammography, BMI, family breast cancer, mammographic device, season of the year of mammography, and age at menarche

^2^ likelihood ratio test (adjusted model)
