# Supplementary material for: Cadmium and volumetric mammographic density: A cross-sectional study in Polish women
Source: PLoS One. 2020 May 20;15(5):e0233369. doi: 10.1371/journal.pone.0233369 (PMC7239444; doi:10.1371/journal.pone.0233369)
Supplement: S4 Table — (DOCX) [file pone.0233369.s004.docx]

S4_Table. Association between cadmium concentration creatinine adjusted in urine and percent volumetric mammographic density and fibroglandular tissue volume by menopausal status

|  | Pre-menopausal  N=116 | | | Post-menopausal  N=402 | | | p-heterogeneity^3^ |
| --- | --- | --- | --- | --- | --- | --- | --- |
|  | β (95%Confidence interval) | | | β (95%Confidence interval) | | |  |
|  | unadjusted | Adjusted^1^ | Adjusted^2^ | unadjusted | Adjusted^1^ | Adjusted^2^ |  |
| Percent volumetric mammographic density | -0.085 (-0.249,0.078) | -0.078 (-0.232, 0.076) | -0.017 (-0.174, 0.140) | -0.019 (-0.103, 0.065) | **-0.086 (-0.159, -0.016)** | **-0.078 (-0.151, -0.004)** | 0.373^1^ |
| Fibroglandular tissue volume | -0.023 (-0.174,0.128) | -0.020 (-0.172,0.132) | 0.006 (-0.153,0.164) | -0.045 (-0.118,0.027) | -0.026 (-0.097,0.045) | -0.020 (-0.094,0.054) | 0.782^1^ |

^1^ Adjusted for age at mammography, BMI, family breast cancer, mammographic device, season of the year of mammography, and age at menarche

^2^ Adjusted for age at mammography, BMI, family breast cancer, mammographic device, season of the year of mammography, age at menarche and smoking

^3^ likelihood ratio test
