# Supplementary material for: Cadmium and volumetric mammographic density: A cross-sectional study in Polish women
Source: PLoS One. 2020 May 20;15(5):e0233369. doi: 10.1371/journal.pone.0233369 (PMC7239444; doi:10.1371/journal.pone.0233369)
Supplement: S5 Table — (DOCX) [file pone.0233369.s005.docx]

S5_Table. Association between cadmium concentration creatinine adjusted in urine and percent volumetric mammographic density and fibroglandular tissue volume by family history of breast cancer

|  | Family history of breast cancer | | | | | | p-heterogeneity^3^ |
| --- | --- | --- | --- | --- | --- | --- | --- |
|  | No  N=412 | | | Yes  N=53 | | |  |
|  | β (95%Confidence interval) | | | β (95%Confidence interval) | | |  |
|  | unadjusted | Adjusted^1^ | Adjusted^2^ | unadjusted | Adjusted^1^ | Adjusted^2^ |  |
| Percent volumetric mammographic density | -0.021 (-0.105,0.063) | -0.063 (-0.134, 0.008) | -0.052 (-0.126,0.022) | **-0.246 (-0.487,-0.004)** | **-0.342 (-0.594,-0.089)** | **-0.370 (-0.645,-0.094)** | 0.288^1^ |
| Fibroglandular tissue volume | -0.040 (-0.112,0.032) | -0.007(-0.078,0.064) | 0.005 (-0.069,0.078) | -0.211(-0.464,0.042) | -0.120(0.409,0.168) | -0.110 0(-0.425,0.206) | 0.587^1^ |

^1^ Adjusted for age at mammography, BMI, mammographic device, season of the year of mammography, and age at menarche

^2^ Adjusted for age at mammography, BMI, mammographic device, season of the year of mammography, age at menarche and smoking

^3^ likelihood ratio test
