# Supplementary material for: Cadmium and volumetric mammographic density: A cross-sectional study in Polish women
Source: PLoS One. 2020 May 20;15(5):e0233369. doi: 10.1371/journal.pone.0233369 (PMC7239444; doi:10.1371/journal.pone.0233369)
Supplement: S6 Table — (DOCX) [file pone.0233369.s006.docx]

S6_Table. Association between cadmium concentration creatinine adjusted in urine and percent volumetric mammographic density and fibroglandular tissue volume by age groups

|  | ≤50  N=85 | | >50 - ≤55  N=211 | | >55  N=221 | | p-heterogeneity^2^ |
| --- | --- | --- | --- | --- | --- | --- | --- |
|  | β (95%Confidence interval) | | β (95%Confidence interval) | | β (95%Confidence interval) | |  |
|  | unadjusted | Adjusted^1^ | unadjusted | Adjusted^1^ | unadjusted | Adjusted^1^ |  |
| Percent volumetric mammographic density | -0.104 (-0.296,0.088) | -0.122 (-0.290,0.046) | 0.064 (-0.062,0.191) | 0.031 (-0.089,0.151) | -0.064 (-0.167,0.039) | -0.130 (-0.226,-0.033) | 0.512^1^ |
| Fibroglandular tissue volume | 0.066 (-0.099,0.231) | 0.068 (-0.119,0.254) | -0.041 (-0.153,0.072) | 0.031 (-0.091,0.152) | -0.047 (-0.138,0.044) | -0.046 (-0.136,0.045) | 0.274^1^ |

^1^ Adjusted for age at mammography (continuous), BMI, family breast cancer, mammographic device, season of the year of mammography, and age at menarche

^2^ likelihood ratio test (adjusted model)
